# Associations between Preschool Teachers’ Food-Based Learning Frequency, Level of Personal Priority and Identified Resources and Challenges: A Needs Assessment

**DOI:** 10.3390/nu16132140

**Published:** 2024-07-04

**Authors:** Jessica Resor, Jocelyn B. Dixon, Qiang Wu, Archana V. Hegde, Tammy D. Lee, L. Suzanne Goodell, Lucía I. Méndez, Valerie Jarvis McMillan, Virginia C. Stage

**Affiliations:** 1 Department of Human Development and Family Science, East Carolina University, Greenville, NC 27858, USA; resorj21@ecu.edu (J.R.); hegdea@ecu.edu (A.V.H.); 2 Department of Agricultural and Human Sciences, North Carolina State University, Raleigh, NC 27695, USA; jocelyn_dixon@ncsu.edu; 3 Department of Public Health, East Carolina University, Greenville, NC 27834, USA; wuq@ecu.edu; 4 Department of Mathematics, Science, and Instructional Technology Education, East Carolina University, Greenville, NC 27858, USA; leeta@ecu.edu; 5 Department of Food, Bioprocessing & Nutrition Sciences, North Carolina State University, Raleigh, NC 27695, USA; suzie_goodell@ncsu.edu; 6 Department of Communication Sciences and Disorders, University of North Carolina Greensboro, Greensboro, NC 27412, USA; limendez@uncg.edu; 7 Department of Family and Consumer Services, North Carolina Agricultural and Technical State University, Greensboro, NC 27412, USA; vmcmilla@ncat.edu

**Keywords:** food-based learning, nutrition education, professional development, head start, preschool, early childhood education

## Abstract

Food-based learning (FBL) is the use of food as a teaching tool in the classroom, which can expose children to healthy foods to improve preference and consumption. However, more research is needed on the use and perception of FBL in the Head Start (HS) preschool classroom. In an online survey, we explored associations between North Carolina HS teachers’ (*n* = 168) experiences (e.g., resources, challenges, needs, and preferences) with FBL, how frequently teachers implemented it, and how much they prioritized it. We used frequencies and chi-square tests of independence to assess associations between study variables. Teachers reported using FBL regularly with access to FBL resources (e.g., books and center play materials) and experiencing challenges (e.g., lack of funding and material resources). Teachers partnered with parents and farmers markets and expressed a need for additional FBL professional development. Our needs assessment findings revealed specific resources, challenges, and perceptions significantly associated with how often teachers used FBL and their priority level. Additional research should investigate how to alleviate FBL challenges and strategies to create policy and environmental changes that facilitate early FBL.

## 1. Introduction

The consumption of healthy foods throughout the first five years of life is lowest during the preschool years (ages 3–5), with 27% of children not consuming vegetables daily [1]. Such a decline may be partially related to children’s neophobia, “fear of the new”, which biologically peaks during preschool years [2]. Positively impacting preschool children’s establishment of healthy food-related and eating behaviors is critical because the dietary patterns children adopt at this age can impact their risk of developing diet-related diseases into adulthood [2,3,4,5,6]. Impacting health behaviors of children from low-resource backgrounds is especially important as these children are at higher risk for low fruit and vegetable consumption [7,8] and may have less access to healthy, affordable food and safe areas for recreational play [9].

Prior research suggests that preschool children in the United States spend more than 30 h a week and consume half or more of their daily dietary intake at preschool, making preschool teachers an important partner in increasing children’s exposure to healthy foods [10]. Over 1 million children from low-resource backgrounds attend Head Start, the federally funded preschool program, which strives to meet children’s nutritional, social, developmental, and academic needs [11]. Head Start programs are required to participate in the Child and Adult Care Food Program (CACFP), which guarantees children access to healthy foods, such as fruits and vegetables, as part of school meals and snacks [12]. Head Start’s participation in CACFP not only increases children’s exposure to healthy foods [13,14] but can also support food-based learning (FBL).

Food-based learning is defined as “the use of healthy food as a teaching tool to provide repeated exposures to healthy foods to improve children’s dietary behaviors and academic learning related to knowledge (e.g., science, mathematics, literacy) and skills (e.g., gross motor, fine, physical)” [15]. Food-based learning can occur both inside and outside the mealtime environment [16] and supports children’s development of healthy dietary behaviors [13,16,17]. This early and repeated exposure to healthy foods is critical as prior research suggests that repeated exposure is the most effective method to impact children’s preference and consumption of healthy foods [18,19]. Using the strategy of repeated exposure, FBL allows children to explore healthy foods multiple times in multiple forms to increase familiarity with a specific food [18,20].

Prior studies have demonstrated FBL’s ability to impact preference for and consumption of healthy foods [21]. In a study conducted by Bayles and colleagues, preschool children participated in seven integrative FBL activities (e.g., science, mathematics, literacy) over 4 months in Head Start classrooms [13]. Each activity ended with an opportunity for children to taste the vegetables. Outcomes of the intervention revealed children in the intervention group consumed significantly more carotenoid-rich fruits and vegetables than the control group over an extended period. Another study explored the effects of the program Together, We Inspire Smart Eating (WISE), an 8-month nutrition education curriculum that exposes children to fruits and vegetables through weekly hands-on, integrative FBL activities [16,21,22]. For example, in one of the integrative FBL activities teaching the mathematical concepts of patterns, children organized skewers of tomatoes, spinach, and cheese [16,21,22]. Children in the intervention group had significantly higher fruit and vegetable intake at post-test compared with baseline according to parent surveys [16].

Head Start teachers are familiar with FBL through their training and work experience. As Head Start strives to support children’s nutritional needs [11], teachers are typically expected to be aware of child feeding practices and build a healthy eating environment during family-style meals where children and teachers eat together [23]. While previous studies have suggested FBL is effective, Head Start teachers still report challenges to FBL in the classroom, such as limited time [24] and competing priorities, namely kindergarten readiness [8,24]. The complexities behind implementing FBL into the classroom have been described by Carraway-Stage and colleagues using a theoretical model that outlines various intervening conditions such as resources, funding, individual and administrative priority, and time that impact the use and quality of FBL in the classroom [24]. Head Start teachers and administrators have suggested that many of these challenges to FBL could be addressed by integrating FBL into other learning domains (e.g., science, math, literacy) to impact dietary quality and kindergarten readiness outcomes simultaneously [24].

In addition to these challenges, Head Start teachers may lack training and resources to deliver FBL [24,25]. Few studies have explored what types of training and resources Head Start teachers have available for FBL [26] and how these trainings impact teachers’ FBL practices. A qualitative study in 2016 among Head Starts in North Carolina (NC) revealed that teachers had access to varying levels of material, human, and training resources related to FBL [26]. Teachers identified that additional FBL resources and training would be beneficial [26]. Nevertheless, it is unknown how teachers’ experiences with these resources, trainings, and challenges influence how frequently they do and how highly they prioritize FBL. To date, little has been studied quantitatively about these aspects of FBL in the Head Start classroom. Additionally, outside of resources and challenges, there may be other critical influences at play. For example, how important teachers perceive FBL, and the larger umbrellas of health and nutrition, may influence how often they implement FBL. Prior research acknowledges that competing demands in the classroom, such as other learning domains [8,27], may decrease the priority placed on FBL. Other studies have reported that not having enough time limits teachers’ ability to deliver nutrition-related lessons [24,28]. To this end, teachers’ perceptions and practices may be influenced by their fellow teachers and administration [29,30]. More grassroots or “bottom-up” influences from the center level may drive change in FBL [31,32]. Change may also come “top-down” from the federal or state-level Head Start administration [33,34]. Therefore, it is also important to assess how teachers perceive others’ prioritization of FBL and their perception of the frequency of FBL implementation.

Given this prior research, there is a need to understand the current state of FBL in Head Start from teachers’ perspectives through a needs assessment. A needs assessment on this topic can identify teachers’ needs, assess potential causes of the current FBL state, and establish future priorities and actions [35]. Further, examining the potential significant relationships among these factors would be pertinent to gain a more robust picture of FBL in the Head Start preschool classroom. Guided by Carraway-Stage’s theoretical model [24], this needs assessment study aimed to examine NC Head Start teachers’ experiences with FBL and what influences their frequency of implementing FBL in their classrooms.

## 2. Materials and Methods

### 2.1. Study Design

This study was part of a larger mixed methods cross-sectional study conducted across NC to assess the specific needs and resources of Head Start programs to inform the development of the teacher professional development program, Preschool Education in Applied Science (PEAS) Institute for Early Childhood Teachers [36]. The overarching goal of PEAS is to (1) build teachers’ science teaching efficacy and pedagogical knowledge and skills and (2) improve children’s science knowledge, development of scientific language, and dietary quality [37].

The present study served as a needs assessment to inform the development of the larger PEAS professional development program. Given prior research on FBL in Head Start centers in NC [24], we developed three research questions (RQs):

1. What experiences have teachers had implementing FBL in their classrooms and engaging in related professional development? (FBL Experiences and Implementation Needs)
2. How is the frequency of teachers’ FBL implementation associated with related professional development needs, available resources, implementation challenges, and their perception of the administration’s priority level for using food as a teaching tool? (Associations with FBL Frequency)
3. How are teachers’ personal priority levels for FBL associated with related professional development needs, available resources, implementation challenges, and their perception of the administration’s priority level for using food as a teaching tool? (Associations with FBL Personal Priority Level)

Based on previous research, we expected that both of our variables of interest (FBL Frequency and FBL Personal Priority Level) would be positively associated with professional development needs, available resources, and perception of the administration’s priority level. We expect that FBL Frequency and FBL Personal Priority Level would be inversely associated with implementation challenges.

Table 1 provides a brief overview of our research questions and analysis plan. The study was conducted in accordance with the rules of the Declaration of Helsinki [38] and underwent an expedited review by the Institutional Review Board at East Carolina University, which approved all study protocols and materials (UMCIRB #18-002749).

### 2.2. Data Collection Procedures

We conducted the study from September 2020 to March 2021 using a purposive sample of NC-based Head Start lead and assistant teachers aged 18 years or older. We identified 52 NC-funded Head Start agencies using a list of all NC agencies listed on Head Start’s website in the Fall of 2020 [39]. We contacted each agency’s education managers or program directors by phone to provide information about the study and ask permission to communicate via email with their teachers. One agency was excluded because it primarily served migrant families and was not open during the winter/spring. Head Start staff chose to either forward our recruitment email to teachers or send us a list of teachers’ emails so we could email teachers directly. Teachers were invited to participate by email, which included study information, the informed consent form, and a link to the survey. Participants provided electronic written informed consent and could skip any questions or stop participating at any time. We administered the anonymous survey using Research Electronic Data Capture (REDCap), a secure, HIPPA-compliant, web-based software platform designed to support data capture for research studies, providing (1) an intuitive interface for validated data capture; (2) audit trails for tracking data manipulation and export procedures; (3) automated export procedures for seamless data downloads to common statistical packages; and (4) procedures for data integration and interoperability with external sources [40,41]. Participants were not given a deadline to complete the survey but were informed that they would be eligible to be entered into a drawing for a USD 95 gift card if the survey was completed by the provided date. The monetary amount was chosen to increase participants’ motivation to complete the survey. Participants were followed up with via email twice a week if they had an incomplete submission to further encourage survey completion.

To collect geographically diverse data (mountains, piedmont, coastal), we visualized the distribution of data on a state map and discussed it as a team weekly. When clusters of participants formed, we focused the next week of recruitment on a different area of the state. For example, if we received an influx of data from the Piedmont region, the next week we would focus recruitment on programs in the coastal or mountain regions of the state instead by reaching back out to Education Managers to request they resend the recruitment email to teachers or following up with teachers via email. Seventeen of the fifty-two agencies responded to the initial communication (32.7% agency response rate). North Carolina Head Start centers often follow their public-school counterparts’ academic calendars. During the time of this study (2020–2021), many public schools were closed due to COVID-19 [42]. It is likely that Head Start agencies were also closed or providing virtual education to children making it difficult to communicate with program administrators and staff [43], which impacted our response rate.

### 2.3. Quantitative Survey and Study Variables

#### 2.3.1. Survey Overview and Development

The online survey consisted of 78 items, 11 of which assessed Head Start teachers’ experiences incorporating FBL experiences in the classroom, specifically related to frequency, challenges, resources, priority level, and training. We provided the following definition of FBL in the survey so that participants knew to what we were referring: “Food-based Learning is defined as using food as a hands-on tool to teach children science including but not limited to gardening and nutrition (e.g., exposing children to healthy foods and discussing how foods help the body grow and be healthy)”. Specific survey items were researcher-developed or adapted to address findings from previous research studies [24,44,45]. As previously mentioned, our survey development was also informed by Carraway-Stage et al.’s theoretical model [24] such that our quantitative items modeled their qualitative items. Other questions in the survey focused on science education, science talk, demographics, and COVID-19; findings are reported elsewhere [46]. Importantly, while data was collected during the beginning of the COVID-19 pandemic, we asked participants to answer questions based on their pre-pandemic practices. Specific questions at the end of the survey allowed participants to reflect on how COVID-19 had impacted their classrooms. Data about COVID-19 is not reported in this manuscript (currently unpublished). The survey is available upon request; please contact the corresponding author.

We took steps to cognitively evaluate survey items with experts and community members similar to study participants. The survey underwent face validation with field experts and teachers before beginning the study. We provided the survey to one Registered Dietitian and one early childhood expert, both of whom were familiar with the subject matter, to review survey questions and assess whether questions effectively addressed the topic. Secondly, the survey was cognitively evaluated during 30 min phone interviews with 2 preschool teachers and 1 administrator recruited by a faculty member from the Department of Human Development and Family Sciences at East Carolina University. Two members of the research team conducted cognitive interviews over the phone to garner feedback from participants on a question-by-question basis [47,48]. Data we collected during cognitive evaluations were not used in the analysis, rather participants were encouraged to provide feedback on areas of confusion, clarification, or general edits to the survey that they deemed beneficial. Results from cognitive interviews did not yield any major concerns with survey items, only minor changes were made to lengthy sentences or unclear wording (e.g., “you”, “their”, etc.). Participants received a USD 10 gift card as compensation for their time. For readability below, variables are listed in bold the first time they are used, and response options are presented as italicized in this text.

#### 2.3.2. Demographic Variables

Participants self-reported demographic data, which was collected to describe the sample, including age, role, education, work experience, gender, race, and ethnicity. Participants open-endedly reported their gender. Teachers self-reported race and ethnicity from a list including *White* or *European American*, *non-Hispanic*; *Latino(a)*, or *Spanish*; *Black* or *African American*, *non-Hispanic*; *Asian* or *Asian American*, *non-Hispanic*; *American Indian* or *Alaskan Native*, *non-Hispanic*; *Middle Eastern* or *North African*; *Native Hawaiian* or *Pacific Islander*; *Multiethnic*; or *other (specify)*. Participants were allowed to select multiple responses to accurately reflect their self-affiliation. The demographic survey followed the US Office of Management and Budget protocols which guide the collection of race and ethnicity data in the US [49,50].

#### 2.3.3. Variables for RQ1 (FBL Experiences and Implementation Needs)

Teachers quantified how often (**frequency**) they implement FBL in their classroom (6 points: Very Often (daily), Regularly (2–4/week), Sometimes (weekly), Rarely (monthly), Almost Never (less than monthly), or None of the Above). We also asked teachers to indicate the types of **FBL resources** available to them from twelve options (e.g., curricular resources, materials for center play, perishable items, etc.) (Available or Not Available). Since many Head Start agencies partner with community organizations, we also asked teachers to identify **partnerships** they utilize to further engage children in FBL from a list of options (e.g., extension, libraries, farmer’s market, etc.) (Check box if used for FBL). To understand how teachers chose FBL activities, we asked teachers to rate the most important **characteristics** when selecting an activity or curricular resource for teaching FBL (e.g., cost, structure/organization of content, ease of use, inclusivity, length, cultural appropriateness, other, or none of the above) (Important, Somewhat Important, Not Important, or I don’t know). Throughout the survey teachers indicated **facilitators and challenges** to using FBL in their classrooms through a list of options (Mark All that Apply). Teachers described what **FBL professional development**, if any, their program required during their first year at Head Start from 7 options (Mark All That Apply), with one of the options asking about reviewing Head Start’s policies on FBL. Teachers also described their **motivations** to participate in professional development. Lastly, teachers were asked to rate the **priority of FBL** in the Head Start environment, both personally and their perception of its priority to others (e.g., fellow teachers, Head Start administrators, and families) (Not at All Important, Not Very Important, Fairly Important, Very Important, or Extremely Important). Teachers could provide any additional information in an open-ended text box at the end of the survey.

#### 2.3.4. Variables for RQs 2 (Associations with FBL Frequency) and 3 (Associations with FBL Personal Priority Level)

For **teachers’ FBL frequency (RQ2)**, we asked teachers on average how frequently do you use FBL to support science learning in their classroom (6 points: *Very Often (daily)*, *Regularly (2–4/week)*, *Sometimes (weekly)*, *Rarely (monthly)*, *Almost Never (less than monthly)*, or *None of the Above*). For **teachers’ personal priority (RQ3)**, we asked teachers to rate the priority they place on FBL in their classroom (5-point scale: *Not at All Important*, *Not Important*, *to Fairly Important* vs. *Very Important* vs. *Extremely Important*).

The following groups of variables, namely FBL professional development needs, available FBL resources, FBL challenges, and administrator’s priority for FBL, were used for RQs 2 and 3. For **FBL professional development needs**, we asked teachers to think of what level of professional development need they have regarding these 5 types: Material Resources, Curricular Resources, Technological Resources, Periodic Training, and Regular Mentoring/Coaching (3-point scale: *No Need at All*, *Some Level of Need*, and *High Level of Need*).

For **available FBL resources**, we asked teachers to report what of these 12 resources were available to them during the last year to teach FBL in their classrooms: A Specific Curricular Resource, Games, Educational Posters, Books, Computer Software, Music, Videos, Materials for Center Play, Refrigerator for Perishable Items, Additional Staff Support to Help with Hands-on Activities, Funds to Support Purchasing New Supplies Needed for New Activities, and Funds to Support Field Trips (*Available* or *Not Available*).

For **FBL challenges**, we asked teachers if they experienced any of the following 9 challenges: Lack of Money for Additional FBL Materials, Lack of Expertise to Provide Age-appropriate Education in this Area, Lack of Human Resources to Support Activities in this Area, Lack of Material Resources, Other Areas in Program Have Higher Priority, Lack of Time in Schedule to Increase the Amount of Education on This Topic, Lack of Knowledge About How to Integrate, Children Would Not Be Interested in Spending More Time Focused on This Topic, and Parents Would Not Support the Idea of Children Spending More Time Focused on This Topic (*Experienced* or *Not Experienced*).

To assess **teachers’ perception of administrator’s priority for FBL**, we asked teachers to rate what level of priority they perceived these 3 levels of administration (i.e., center, state, and federal Head Start administration) placed on FBL (5-point scale: *Not at All Important*, *Not Important*, to *Fairly Important* vs. *Very Important* vs. *Extremely Important*).

### 2.4. Data Analysis Procedures

We used mean and standard deviation or frequency (%) to describe demographics and ratings on each topic. To assess the association between Teachers’ FBL Implementation Frequency with our four categories of interest (i.e., FBL professional development needs, FBL resources, FBL challenges, and Administrator’s perceived priority), we conducted chi-square tests of independence. Then, to assess the association between Teachers’ Personal Priority Level for FBL, respectively, with our four categories of interest, we conducted another set of chi-square tests of independence. We reported effect size for significant associations using Cramer’s V with 0.2 < *V* ≤ 0.6 being moderate [51,52].

In the chi-square tests, for analytical purposes, teachers’ FBL frequency was divided into two groups: *Regularly (2–4/week)* or *More* vs. *Sometimes (weekly)* or *less*. *Regularly* or *more* combined the response options of *Very often* and *Regularly*. *Sometimes* or *less* combined the response options of *Almost never*, *Rarely*, and *Sometimes*. Teachers’ personal FBL priority was divided into three response groups for analytical purposes: *Not at All Important*, *Not Important* to *Fairly Important* vs. *Very Important* vs. *Extremely Important*. Our other variables of interest were teachers’ FBL professional development needs (5 variables divided into 3 response groups for analytical purposes: *No Need at All* vs. *Some Level of Need* vs. *High Level of Need*), perceived administrators’ FBL priority (3 variables, divided into 3 response groups: *Not at All Important*, *Not Important*, to *Fairly Important* vs. *Very Important* vs. *Extremely Important*), available FBL resources (12 variables, *Available* or *Not Available*), and FBL challenges (9 variables, *Experienced* or *Did Not Experience the Challenge*). All statistical analyses were conducted using SPSS (version 28.0, IBM Corp, Armonk, NY, USA, 2017). A significance level of *p* < 0.05 was used for all statistical tests.

Given that participants could skip any question, there was a small amount of missing data (typically ranging from 0–12 missing responses (0–7%) per measure). Our analytical sample size is reported throughout the results and in tables when appropriate. Missing data were handled with pairwise deletion.

## 3. Results

**Figure 1:**
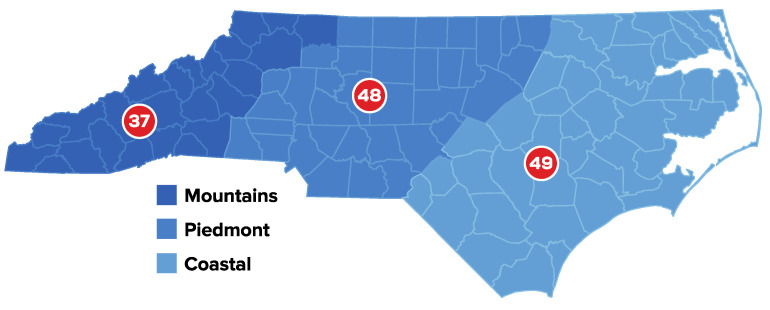
Teachers’ participation (*n* = 134; 34 did not report location) by North Carolina regions participating in the survey regarding their experiences with food-based learning in Head Start.

A total of 168 teachers responded to the survey. Participants were diverse from both large and small agencies, lead and assistant teachers, and all three regions of the state: Mountain (31.0%), Piedmont (26.8%), and Coastal (29.8%) (Figure 1). All but two participants identified as female (92.3%, *n* = 155). Regarding teachers’ roles, over sixty percent of the sample were in the role of a lead teacher (62.5%, *n* = 105), followed by assistant teachers (24.4%, *n* = 41) and others who considered themselves as other types of teachers (e.g., floater teacher) (8.9%, *n* = 15). Four participants indicated different roles: Head Start Program Director, Health/Nutrition Manager, Center Director, and one other specified as a teacher and nutrition assistant. Participants self-identified their race and could select all options that applied. The sample was 50.6% Black or African American (non-Hispanic), 38.7% White or European American (non-Hispanic), 5.4% Latino/Latina or Spanish, and 1.2% multiethnic. No participants identified as Asian, American Indian, or Alaskan Native, Middle Eastern or North African, or Native Hawaiian or Pacific Islander. Participants were an average age of 43 (*SD* = 11.5, range = 23–67) years at the time of the study. Teachers’ educational levels varied: 50.6% held a 4-year degree, 31% had a 2-year associate degree or less, and 16.1% had some graduate coursework or higher. Seventy percent of all teachers’ highest degrees were in the field of early childhood education. Teachers had worked at Head Start for an average of 8.57 years (*SD* = 7.78, range = 1–36); however, the majority (71.4%) of surveyed teachers also had experience working in other preschool settings outside of Head Start.

### 3.1. Findings on RQ1 (FBL Experiences and Implementation Needs)

To address RQ1, we asked teachers about their use of FBL, resources, challenges, and needs and preferences for FBL and professional development. Table 2 presents the frequencies of all study variables.

#### 3.1.1. Frequency of Implementing FBL

When asked how frequently, on average, they use FBL to support science learning in their classrooms, more than half of the study sample (*n* = 102, 60.7%) reported using FBL in their classroom regularly (2–4/week; *n* = 40, 23.8%) to very often (daily; *n* = 62, 36.9%). About a quarter (*n* = 39, 23.8%) reported using FBL sometimes (weekly). Only a small number of teachers (*n* = 21, 12.5%) reported rarely (1/month; *n* = 13, 7.7%) or almost never (<1/month; *n* = 8, 4.8%) using FBL in their classroom. Consequentially, and for analytical purposes, this information means that most participants reported using FBL *Regularly* or *more* (60.7%), and approximately a third (35.7%) reported using FBL *Sometimes* or *less*.

#### 3.1.2. Resources and Partnerships

Teachers reported that the most common FBL resources available to them over the past year were materials for center play (*n* = 156, 92.9%) and books (*n* = 155, 92.3%). Access to refrigeration to store perishable items (*n* = 112, 66.7%), funds to purchase supplies for activities including perishable items (*n* = 100, 59.5%), and curricular resources (*n* = 110, 65.5%) were largely accessible but reportedly less available.

Teachers reported they partnered with a variety of outside organizations to provide FBL in their classrooms during the past year. The top partnerships were with parents/guardians (*n* = 32, 19%), local farmers’ markets (*n* = 29, 17.3%), Special Supplemental Nutrition Program for Women, Infants, and Children (WIC) (*n* = 29, 17.3%), local grocery stores (*n* = 27, 16.1%), Cooperative Extension/Expanded Food and Nutrition Program (EFNEP) (*n* = 26, 15.5%), local food banks (*n* = 26, 15.5%), Registered Dietitians (*n* = 24, 14.3%), and the Supplemental Nutrition Assistance Program Education (SNAP-Ed) (*n* = 21, 12.5%). Approximately one in five teachers (*n* = 38, 22.6%) reported no community partnerships to support FBL efforts in the last year.

#### 3.1.3. Challenges, Needs, and Preferences to FBL and Professional Development

When selecting FBL activities, teachers considered cultural appropriateness (*n* = 132, 78.6%), having all the materials needed for implementation (*n* = 127, 75.6%), and clear directions for ease of implementation (*n* = 111, 66.1%) as most important. Overall, teachers personally felt FBL was either extremely or very important (*n* = 126, 75.0%). Very few teachers (*n* = 5, 3.0%) felt FBL was not important. However, teachers faced a variety of challenges in implementing FBL in their classrooms. The most frequently reported barrier was funding to purchase perishable items (*n* = 72, 42.9%), followed by a lack of material resources (*n* = 49, 29.2%) or human resources (*n* = 46, 27.4%) to support FBL. A group of teachers (*n* = 24, 14.3%) reported facing no challenges to FBL. Despite the reported challenges, teachers felt that their center (*n* = 111, 66.1%), state (*n* = 109, 64.9%), and federal (*n* = 117, 69.7%) Head Start administration prioritized FBL as extremely or very important.

Regarding FBL training, we asked teachers how their program trained them on FBL when they were in their first year as a new hire. Some teachers reported they received training on how to implement FBL by attending workshops or training sessions (*n* = 69, 41.1%) or through collaboration with other teachers (*n* = 73, 43.5%). Conversely, nearly one in four teachers (*n* = 40, 23.8%) reported they received no FBL training, and less than a quarter (*n* = 37, 22.0%) of all teachers reported being required to review their program’s written FBL policies. After reflecting on their prior training in FBL, 75% of teachers felt that they either had some (*n* = 78, 46.4%) or a high (*n* = 48, 28.6%) level of need for FBL professional development resources. Similarly, 73.2% of teachers felt they had some (*n* = 99, 58.9%) or high (*n* = 24, 14.3%) level of need for professional development in FBL. Fewer teachers (*n* = 17, 10.0%) felt that they did not need FBL professional development.

Teachers stated they were motivated to participate in professional development due to a desire to grow and improve job performance as an early childhood professional (*n* = 155, 92.3%), stay up to date with best practices (*n* = 139, 82.7%), in response to passion for their job (*n* = 135, 80.4%), better prepare children for kindergarten (*n* = 133, 79.2%), and to meet children’s overall needs (*n* = 130, 77.4%).

### 3.2. Findings on RQ2 (Association with FBL Frequency)

To address RQ2, we examined potential associations between teachers’ FBL frequency and our variables of interest from these categories: FBL Professional Development Needs, Available FBL Resources, FBL Challenges, Perceived Administrator’s Priority. The results of each of the chi-square tests are presented in Table 3.

None of the five professional development needs variables were significantly associated with teachers’ FBL frequency. A specific curricular resource was the only item from the twelve available resources to have a statistically significant relationship with teachers’ FBL frequency, *ꭓ*^2^ (1) = 9.14, *p* = 0.002, *V* = 0.25. The effect size indicates that these two variables are moderately and positively associated indicating teachers with access to a specific curricular resource reported a higher frequency of FBL in the classroom (69.4%) than those without (30.6%). Two of the nine items from FBL challenges had a statistically significant relationship with teachers’ FBL frequency: lack of money, *ꭓ*^2^ (1) = 4.92, *p* = 0.02, *V* = −0.17 and lack of materials, *ꭓ*^2^ (1) = 7.83, *p* = 0.005, *V* = −0.22. The effect size indicates that lack of money and FBL frequency are weakly and inversely associated. Teachers who reported lack of money as a challenge to FBL reported a lower frequency of FBL in the classroom (53.2.%) than those who did not (46.8%). The effect size for lack of materials indicates that the two variables are moderately and inversely associated meaning that teachers who did not report lack of materials as an FBL challenge reported a higher frequency of FBL in the classroom (70.2%) than those who did (29.8%). One of the three teachers’ perceptions of administration FBL priority level was significantly associated with teachers’ FBL frequency: federal Head Start, *ꭓ*^2^ (2) = 7.43, *p* = 0.02, *V* = 0.22. The effect size indicates that the two variables are moderately and positively associated meaning those who perceived federal Head Start administration to place higher priority on FBL reported higher frequency of FBL in the classroom. About three-quarters (74.6%) of teachers who perceived the federal Head Start administration’s priority to be very important reported higher FBL frequency compared with the 55.8% of teachers who perceived the federal Head Start administration’s priority as extremely important.

### 3.3. Findings on RQ3 (Associations with FBL Personal Priority Level)

To address RQ 3, we examined potential associations between teachers’ personal priority for FBL and our variables of interest (i.e., FBL Professional Development Needs, Available FBL resources, FBL challenges, Administrators’ perceived priority). The results of each of the chi-square tests are presented in Table 3.

One professional development needs variable, curricular resources, was significantly associated with teachers’ personal priority for FBL, *ꭓ*^2^ (4) = 11.29, *p* = 0.02, *V* = 0.19. The effect size indicates a weak and positive association. Four FBL available resources were significantly associated with teachers’ personal priorities for FBL. A specific curricular resource was significantly associated with teachers’ personal priority, *ꭓ*^2^ (2) = 10.22, *p* = 0.006, *V* = 0.26. Books were significantly associated with teachers’ personal priority, *ꭓ*^2^ (2) = 6.94, *p* = 0.03, *V* = 0.21. Computer software was significantly associated with teachers’ personal priority, *ꭓ*^2^ (2) = 8.92, *p* = 0.01, *V* = 0.25. Additional staff support to help with hands-on activities was associated with teachers’ personal priority, *ꭓ*^2^ (2) = 8.46, *p* = 0.01, *V* = 0.24. The effect sizes for these four available resources indicate that these associations were positively and moderately associated such that teachers rated their personal priority as higher when they had these resources available. None of the nine FBL challenges were significantly associated with teachers’ personal priorities. All three of the teachers’ perceptions of administration FBL priority level (center, state, and federal) were significantly associated with teachers’ personal priority. Center administration was significantly associated with personal priority, *ꭓ*^2^ (4) = 98.27, *p* < 0.0001, *V* = 0.55. State administration was significantly associated with personal priority, *ꭓ*^2^ (4) = 80.48, *p* < 0.0001, *V* = 0.50. Lastly, federal Head Start administration was significantly associated with personal priority, *ꭓ*^2^ (2) = 74.94, *p* < 0.0001, *V* = 0.49. The effect sizes for administration indicate that these associations are positively and moderately associated, such that teachers who indicated that FBL was a higher priority for center, state, and federal Head Start administrators reported FBL as a higher personal priority.

## 4. Discussion

In this needs assessment, we quantitatively examined NC Head Start teachers’ FBL Experiences and Implementation Needs (RQ1). We also examined Associations with FBL Frequency (RQ2) and Associations with FBL Personal Priority Level (RQ3).

Our findings related to RQ1 (FBL Experiences and Implementation Needs) raise important points about guidance and access to resources, professional development opportunities, and policy issues. Teachers reported frequently using FBL as a teaching method in their preschool classrooms and held FBL as an important personal priority. Notably, 59.5% of teachers appeared to have adequate access to material resources to support FBL (e.g., books, center time materials, posters), except for funding for perishable foods. While teachers have access to several resources, they may need guidance on selecting high-quality resources and curricula to use. Early childhood educators, in general, have limited experience with food and nutrition education [53], which may negatively impact their ability to select high-quality FBL resources and provide quality experiences to young children [15,24,54].

Despite implementing and prioritizing FBL, teachers indicated a need for maximizing access to resources and partnerships, minimizing challenges, and increasing training on how to design evidence-based FBL activities in the classroom and how to comply with Head Start policies governing FBL in the classroom. Among our sample, 75% of teachers reported some level of need for FBL professional development. Prior research among Head Start teachers also supports the need for additional training to support FBL best practices [15,24,55]. Related to FBL policy, less than a quarter of our sample were required to review their center’s policies and guidelines on FBL within their first year of hire. More often, teachers learned about FBL policy through discussions with other teachers at their centers. While peer mentorship is to be encouraged in the education setting [56], prior research suggests that communication among teachers may lead to misinterpretation and miscommunication of center policies related to FBL [15,45]. For example, in one study, Head Start teachers reported that other teachers in their center told them that they were not allowed to bring any food into the classroom, and then they later found out that the center policy did allow healthy foods to be brought into the classroom [15]. In the same study, two teachers described the same FBL activity, using cereal to practice counting, with one teacher citing the activity as in compliance with their Head Start center’s policy, while the other cited the same activity as out of compliance [15]. Ultimately, the way nutrition and FBL-related policies are communicated to teachers is not clearly understood [24,45]. The lack of understanding of how policies are communicated is concerning because teachers’ perception of FBL policy directly impacts their utilization of FBL [45]. Prior research suggests that as teachers perceive a higher level of policy regulation in the classroom (e.g., more restrictive policies about bringing food into the classroom, cooking procedures, and materials allowed), the less frequently they use FBL [45]. Communicating about the center’s FBL policies early and often could increase a culture of compliance and understanding. The administration could set an explicit goal to review the FBL policy with teachers at monthly staff meetings.

Our findings related to RQ2 (Associations with FBL Frequency) suggest that while perceived professional development needs and frequency of FBL implementation are not related, there may be other specific resources and challenges at play. The relationship between teachers’ perceived professional development needs and the frequency of implementing FBL in their classrooms was not significant, meaning that teachers’ frequency of FBL did not depend on their own perceived need for FBL professional development. It is possible, however, that this survey did not capture the “full story” of the relationship between perceived professional development needs since teachers were not asked about their perception of professional development needs within the context of other domains besides FBL (e.g., professional development needs for promoting early literacy, mathematics, etc.). We inquired about teachers’ self-reported needs for professional development in this area. Perhaps professional development needs for FBL are an unfelt or unrecognized need [57] for teachers given the ever-shifting landscape of early childhood care and education and job demands [58,59]. A goal of needs assessments and programming work is to determine felt and unfelt needs and, when appropriate, to facilitate making unfelt needs felt [60]. This is important work because prior research in both preschool and K-12 settings suggests that providing professional development in targeted domains can increase teachers’ frequency of implementing that domain in the classroom [61,62]. For example, Tuttle and colleagues found that teachers who received science professional development increased the frequency with which they used science teaching best practices, such as asking children follow-up questions to think critically, as well as increased the frequency with which science learning appeared in written lesson plans [61]. Therefore, future research is needed to assess teachers’ needs in this area further and explore the relationship between professional development and FBL frequency in the preschool classroom. Future research should also evaluate the impact and outcomes of the specific professional development opportunities teachers attend.

Providing teachers with professional development around FBL is important as it may increase how often they implement FBL in the classroom [61]. More frequent FBL in the classroom is critical as prior research suggests children need 8–15 exposures to increase their liking of a new food [18,19]. While parents and caregivers can contribute to the number of exposures, exposure at home is increasingly difficult as parents may become frustrated with providing foods that are consistently rejected due to children’s neophobia (“fear of the new”) or food waste concerns in families from low-resource backgrounds [2,63,64]. Since 59.3% of preschool children from families with low resources spend over 30 h in childcare a week, where they consume two-thirds of their daily dietary intake [10], Head Start teachers are important partners in increasing children’s exposure to healthy foods using FBL [65,66]. Therefore, increasing teachers’ efficacy for FBL is necessary to promote an environment supportive of teachers’ continued use of FBL [67].

While professional development needs were not significantly related to teachers’ frequency of FBL implementation, lack of money and resources were significantly related to teachers’ frequency of implementing FBL in the classroom. Teachers who reported having access to curricular resources were more likely to report doing FBL with greater frequency in the classroom. Similarly, teachers who had less access to curricular resources reported doing FBL less. Prior research has reported Head Start teachers have used their personal money to purchase food out-of-pocket for FBL experiences in the classroom [24,26]. Teachers purchasing food personally may be a barrier as Head Start teachers may come from low-resource backgrounds themselves, like the children and families they serve [68]. Therefore, funds for FBL are a critical barrier [24,26,68,69]. Furthermore, while we examined several resources and challenges to FBL, there may be other resources and challenges that we did not ask about that may be impacting teachers’ personal priorities and FBL frequencies. Future studies may wish to further specify or disaggregate categories of resources and challenges. Additionally, future research is needed to understand the resources that teachers feel are needed and the policy, system, and environmental changes necessary to assist teachers in implementing FBL more frequently in the classroom.

Findings from RQ3 (Associations with FBL Personal Priority Level) suggest curricular resources and priority levels remain important to the level that teachers themselves place on FBL. Teachers’ need for specific curricular resources was related to the level of priority they personally placed on FBL. Access to specific resources, like curriculum, books, and additional staff, was related to priority level meaning those with access to such items, had a higher level of personal priority for FBL. Teachers in this study felt administrators placed a high priority on FBL in the classroom. Teachers’ personal priorities and their perception of the administrators’ level of priority for FBL were significantly related. In our study, when teachers perceived that administrators prioritized FBL more, they also perceived themselves to prioritize FBL more. This finding is congruent with previous research that found teachers may be more likely to utilize FBL in their classroom when administrators are supportive of teachers spending time on the topic [24]. Similar to teachers modeling the importance of FBL for children in the classroom [15,70], Head Start administration from the center level to the federal level should demonstrate the value they place on FBL to model for teachers. Policy changes and efforts, such as offering PD and providing financial support for personnel and resources, that support more FBL would also show the priority and buy-in from the administration.

Unfortunately, although teachers have reported prioritizing FBL in prior studies, nutrition may not be given the same priority level as other subjects that are more traditionally associated with Head Start’s goal of kindergarten readiness, such as math and literacy [24,45,64]. In addition, Head Start teachers may be overwhelmed by federal teaching requirements, further diminishing the priority placed on perceived “extras” like nutrition [45,64,71]. Thus, time spent on nutrition and food experiences in the classroom may be minimal [64]. Head Start teachers and administrators have hypothesized that integrating FBL into other learning domains could alleviate challenges such as competing priorities while also positively impacting dietary quality and kindergarten readiness outcomes [24,45]. However, teachers may not be knowledgeable about integrating food experiences with other learning domains like science, mathematics, and literacy [15]. For example, in a recent study by Dixon and colleagues, teachers were asked to describe an FBL activity they had carried out in their classroom. Teachers described creating a model of a butterfly using celery and tomatoes during a unit on pollinators [15]. Exposing children to healthy foods outside of the mealtime environment is positive because it allows children to be exposed to healthy foods in a low-pressure environment that encourages children’s exploration using all five senses [14,72,73,74]. However, there is a weak connection between teachers’ design of FBL activity (e.g., making a model butterfly out of celery and tomatoes) and the scientific concept being studied (pollinators). This has led to the use of “disconnected” FBL experiences in the classroom, where food is more often used as art or construction material to illustrate an academic concept, rather than utilizing food as the academic concept (e.g., life sciences) to be studied [15]. Teachers recognize that they need additional training in FBL to design and implement more quality integrative FBL activities [15,75,76]. Emphasizing the potential of FBL to positively impact children’s long-term academic outcomes during FBL trainings that teachers attend may also improve teacher buy-in related to the frequency and personal priority of FBL in the classroom [24,69].

### 4.1. Limitations

This study was conducted with teachers working in Head Start centers in NC. The findings may not be generalizable to other states or representative of the entire United States. We could not calculate the survey’s overall response rates by teachers because we did not know the total number of teachers (potential participants) each center had. However, we made efforts to ensure the survey reached potential participants from various program sizes, roles, and geographic regions by strategically following up with centers and participants who had started the survey. Our efforts and experience appear to be aligned with other survey research during the COVID-19 pandemic, which experienced greater geographical dissemination but lower response rates likely due to survey fatigue and other factors [77]. The timing of the COVID-19 pandemic may have also increased teacher stress and the strain on early childhood education resources [78], which may negatively impact teachers’ FBL implementation. However, teachers were asked with each question to answer questions based on their pre-pandemic classroom practices. While outside of the scope of our study, it will be important to examine in future studies if there are significant changes in teachers’ FBL after the pandemic. Future research could explore teachers’ needs and resources in other states or among other preschool populations. Lastly, we cannot rule out participants providing socially desirable answers. Teachers who were more interested in the topic of FBL may have been more inclined to take the survey.

### 4.2. Implications and Future Research

This study quantitatively explored teachers’ needs and resources for FBL in the preschool classroom and the relationship between these variables and their personal priority and frequency of FBL in the classroom. Understanding which FBL resources teachers already have access to, compared with what they perceive their FBL needs to be, is critical when designing future FBL programming. Funds to purchase food for FBL in the classroom was a significant barrier to teachers. Future research could explore automated and low-cost methods for providing Head Start teachers with healthy foods for FBL. For example, Head Start programs are required to participate in the CACFP, which regulates the foods provided to children during meals and snacks and also allows participating centers to purchase foods for educational purposes [79]. Providing Head Start programs with additional training and support emphasizing how to utilize CACFP funds to support FBL endeavors may decrease challenges to funding FBL. When planned in advance, teachers can coordinate food supplies needed for FBL activities with center directors, who could add these foods to monthly CACFP ordering to reduce out-of-pocket costs for teachers [80]. Partnerships with land grant University Extension programs and SNAP-Ed may also be a source of funding to support FBL through human and material (perishable and nonperishable) resources. In the current study, these federally funded local resources appeared underutilized, pointing to an important opportunity to expand Extension and SNAP-Ed programming in Head Start programs. Finally, future training programs may need to focus on how to support age-appropriate FBL and effectively integrate FBL into other learning domains (e.g., math, science, literacy). Teachers and administrators have identified integrating FBL into other learning domains as a potential solution to address challenges such as limited time and competing priorities in the preschool classroom [24], and additional training is needed to support teachers in this endeavor.

## 5. Conclusions

Effective FBL as a teaching tool can provide structure and opportunity for positively impacting the health and academic outcomes of young children. It is important to understand what teachers need to implement FBL and minimize their challenges. In this study, we conducted a needs assessment on FBL in Head Start through a statewide survey in NC. Teachers reported implementing FBL frequently while navigating challenges and using the resources at their disposal, which is common for teachers to “do more with less” at all levels. While teachers indicated they do need professional development in this area, their professional development needs were not related to how frequently they implement FBL. Certain challenges did impact FBL frequency, which suggests that there are other factors to be explored that influence how often teachers implement FBL. How teachers prioritize FBL and perceive the administration’s prioritization of FBL remains important for teachers and administration to be on the “same page” about FBL efforts. With a current understanding of what resources and challenges NC Head Start teachers face from this study, we call for efforts to review and extend policies and partnerships to Head Start teachers that can provide high-quality materials or funding for FBL. We also call for targeted FBL programming, training, and professional development that can support teachers in successfully and confidently integrating developmentally appropriate FBL into other domains in the preschool classroom. Through these efforts, and with administrators’ support and tangible resources and partnerships, teachers may feel more equipped to deliver FBL to young children, which has the potential to spark lifelong interest and learning in science and influence health and academic outcomes.

## Figures and Tables

**Table 1:** Overview of Research Questions, Variables, and Analysis Plan.

Research Question	Analysis	Rationale	Variable
RQ 1: FBL Experiences and Implementation Needs	Frequencies; Descriptive Statistics	Report and describe experiences and rating on each topic	FBL frequency implementation Partnerships Characteristics of FBL activities Facilitators and Challenges PD experiences and motivations Priority level
RQ 2: Associations with FBL Frequency	Chi-square tests of independence	Determine association between categorical or nominal variables	FBL frequency implication PD needs Available resources FBL challenges Administration priority level
RQ 3: Associations with FBL Personal Priority Level	Chi-square tests of independence	Determine association between categorical or nominal variables	Personal priority level PD needs Available resources FBL challenges Administration priority level

Note: FBL = food-based learning; PD = professional development.

**Table 2:** Frequencies of study variables.

Variable	*n* (%)
FBL Frequency	162 (96.43)
Almost Never (less than monthly)	8 (4.76)
Rarely (monthly)	13 (7.74)
Sometimes (weekly)	39 (23.21)
Regularly (2–4/week)	40 (23.81)
Very Often (daily)	62 (36.9)
Teachers’ Personal Priority for FBL	164 (97.62)
Not important at all to Fairly Important	38 (22.62)
Very Important	87 (51.79)
Extremely Important	39 (23.21)
PD Needs	
Material Resources	155 (89.9)
No need at all	29 (17.26)
Some need	78 (46.43)
High need	48 (28.57)
Curriculum Resources	151 (89.9)
No need at all	32 (19.05)
Some need	84 (50.00)
High need	35 (20.83)
Technology Resources	149 (88.70)
No need at all	37 (22.02)
Some need	71 (42.26)
High need	41 (24.40)
Periodic Training	149 (88.70)
No need at all	26 (15.48)
Some need	99 (58.93)
High need	24 (14.29)
Mentoring/Coaching	141 (83.92)
No need at all	46 (27.38)
Some need	77 (45.83)
High need	18 (10.71)
Administration’s Priority on FBL Center HS Administration	162 (96.42)
Not at all to Fairly Important	51 (30.36)
Very Important	83 (49.40)
Extremely Important	28, (16.67)
State HS Administration	162 (96.4)
Not at all to Fairly Important	53 (31.55)
Very Important	77 (45.83)
Extremely Important	32 (19.05)
Federal HS Administration	158 (94.05)
Not at all to Fairly Important	41 (24.40)
Very Important	65 (38.70)
Extremely Important	52 (30.95)
Available Resources Curricular Resource	156 (92.86)
Not Available	46 (27.38)
Available	110 (65.48)
Games	156 (92.86)
Not Available	35 (20.83)
Available	121 (72.02)
Educational Posters	157 (93.45)
Not Available	31 (18.45)
Available	126 (75.00)
Books	161 (95.83)
Not Available	6 (3.57)
Available	155 (92.26)
Computer Software	143 (85.12)
Not Available	56 (33.33)
Available	87 (51.79)
Music	159 (94.64)
Not Available	29 (17.26)
Available	130 (77.38)
Videos	151 (89.90)
Not Available	24 (14.29)
Available	127 (75.60)
Materials for Center Play	162 (96.42)
Not Available	6 (3.57)
Available	156 (92.86)
Refrigerator for Perishable Items	142 (84.52)
Not Available	30 (17.86)
Available	112 (66.67)
Additional Staff Support	151 (89.90)
Not Available	43 (25.60)
Available	108 (64.29)
Funds to Purchase Supplies	144 (85.7)
Not Available	44 (26.19)
Available	100 (59.52)
Funds for Field Trips	111 (66.07)
Not Available	49 (29.17)
Available	62 (36.90)
FBL Challenges Lack of money for additional FBL materials	167 (99.40)
No	95 (56.55)
Yes	72 (42.86)
Lack of expertise	167 (99.40)
No	138 (82.14)
Yes	29 (17.26)
Lack of human resources	167 (99.40)
No	121 (72.02)
Yes	46 (27.38)
Lack of material resources	167 (99.40)
No	118 (70.24)
Yes	49 (29.17)
Other areas higher priority	167 (99.40)
No	141 (83.93)
Yes	26 (15.48)
Lack of time	157 (93.45)
No	139 (82.74)
Yes	28 (16.67)
Lack of knowledge to integrate	167 (99.40)
No	143 (85.12)
Yes	24 (14.29)
Children not interested in topic	167 (99.40)
No	152 (90.48)
Yes	15 (8.93)
Parents would not support this topic	167 (99.40)
No	152 (90.48)
Yes	15 (8.93)

Note: FBL = food-based learning; PD = professional development; HS = Head Start.

**Table 3:** Chi-squares tests between HS teachers’ FBL frequency and personal priority and study variables.

	FBL Frequency		*ꭓ* ^2^	*p*	Teachers’ Personal Priority for FBL			*ꭓ* ^2^	*p*
	Low FBL Frequency (Weekly or Less)	High FBL Frequency (Regularly 2–4/Week or More)			Not at All to Fairly Important	Very Important	Extremely Important		
Variable	*n* (%)	*n* (%)			*n* (%)	*n* (%)	*n* (%)		
PD Needs									
Material Resources			5.62	0.06				8.21	0.08
No need at all	9 (31.0%)	20 (69.0%)			5 (17.2%)	20 (69.0%)	4 (13.8%)		
Some need	22 (29.7%)	52 (70.3%)			19 (24.7%)	41 (53.3%)	17 (22.1%)		
High need	24 (50.0%)	24 (50.0%)			13 (27.1%)	18 (37.5%)	17 (35.4%)		
Curriculum Resources			2.92	0.23				11.29	0.02 *
No need at all	8 (25.8%)	23 (74.2%)			5 (15.6%)	22 (68.8%)	5 (15.6%)		
Some need	32 (39.5%)	49 (60.5%)			19 (22.9%)	44 (53%)	20 (24.1%)		
High need	16 (45.7%)	19 (54.3%)			13 (37.1%)	10 (28.6%)	12 34.3%		
Technology Resources			0.42	0.81				6.08	0.19
No need at all	12 (33.3%)	24 (66.7%)			7 (18.9%)	22 (59.5%)	8 (21.6%)		
Some need	27 (39.7%)	41 (60.3%)			17 (24.3%)	39 (55.7%)	14 (20.0%)		
High need	15 (36.6%)	26 (63.4%)			11 (26.8%)	15 (36.6%)	15 (36.6%)		
Periodic Training			3.60	0.17				4.18	0.38
No need at all	6 (24.0%)	19 (76.0%)			4 (15.4%)	18 (69.3%)	4 (15.4%)		
Some need	38 (39.6%)	58 (60.4%)			25 (25.5%)	47 (48.0%)	26 (26.5%)		
High need	12 (50.0%)	12 (50.0%)			7 (29.2%)	11 (45.8%)	6 (25.0%)		
Mentoring/Coaching			1.63	0.44				3.23	0.52
No need at all	16 (34.8%)	30 (65.2%)			10 (21.7%)	28 (60.9%)	8 (17.4%)		
Some need	25 (34.3%)	48 (65.8%)			21 (27.6%)	37 (48.7%)	18 (23.7%)		
High need	9 (50.0%)	9 (50.0%)			3 (16.7%)	9 (50.0%)	6 (33.3%)		
Administration’s Priority on FBL									
Center HS Administration			1.12	0.57				98.27	<0.0001 ***
Not at all to Fairly Important	21 (42.9%)	28 (57.1%)			32 (62.8%)	14 (27.4%)	5 (9.8%)		
Very Important	27 (33.7%)	53 (66.3%)			4 (4.8%)	64 (77.1%)	15 (18.1%)		
Extremely Important	11 (39.3%)	17 (60.7%)			1 (3.6%)	8 (28.5%)	19 (67.9%)		
State HS Administration			1.50	0.47				80.5	<0.0001 ***
Not at all to Fairly Important	22 (44.0%)	28 (56.0%)			31 (58.5%)	16 (30.2%)	6 (11.3%)		
Very Important	27 (36.0%)	48 (64.0%)			4 (5.2%)	59 (76.6%)	14 (18.2%)		
Extremely Important	10 (31.3%)	22 (68.7%)			2 (6.2%)	11 (34.4%)	19 (59.4%)		
Federal HS Administration			7.43	0.02 *				74.94	<0.0001 ***
Not at all to Fairly Important	19 (50.0%)	19 (50.0%)			25 (61%)	13 (31.7%)	3 (7.3%)		
Very Important	16 (25.4%)	47 (74.6%)			5 (7.7%)	51 (78.5%)	9 (13.8%)		
Extremely Important	23 (44.2%)	29 (55.8%)			5 (9.6%)	20 (38.5%)	27 (51.9%)		
Available Resources									
Curricular Resource			9.14	0.002 **				10.22	0.006 **
Not Available	25 (56.8%)	19 (43.2%)			17 (37%)	23 (50.0%)	6 (13.0%)		
Available	33 (30.6%)	75 (69.4%)			17 (15.9%)	58 (54.2%)	32 (29.9%)		
Games			2.92	0.09				3.52	0.17
Not Available	17 (50.0%)	17 (50%)			11 (31.4%)	19 (54.3%)	5 (14.3%)		
Available	40 (33.9%)	78 (66.1%)			24 (20.3%)	61 (51.7%)	33 (28%)		
Educational Posters			1.63	0.20				4.44	0.11
Not Available	14 (46.7%)	16 (53.3%)			11 (35.5%)	16 (51.6%)	4 (12.9%)		
Available	42 (34.2%)	81 (65.9%)			25 (20.3%)	65 (52.9%)	33 (26.8%)		
Books			2.44	0.12				6.94	0.03 *
Not Available	4 (66.7%)	2 (33.3%)			4 (66.7%)	2 (33.3%)	0 (0%)		
Available	53 (35.3%)	97 (64.7%)			33 (21.7%)	80 (52.6%)	39 (25.7%)		
Computer Software			3.56	0.06				8.92 *	0.01 *
Not Available	26 (48.2%)	28 (51.9%)			20 (35.7%)	22 (39.3%)	14 (25%)		
Available	27 (32.1%)	57 (67.9%)			12 (14.3%)	47 (55.9%)	25 (29.8%)		
Music			1.11	0.29				0.42	0.81
Not Available	13 (44.8%)	16 (55.2%)			8 (27.6%)	14 (48.3%)	7 (24.1%)		
Available	43 (34.4%)	82 (65.6)%			28 (22%)	67 (52.8%)	32 (25.2%)		
Videos			0.63	0.43				2.01	0.37
Not Available	7 (29.2%)	17 (70.8%)			8 (33.4%)	11 (45.8%)	5 (20.8%)		
Available	46 (37.7%)	76 (62.3%)			25 (20.2%)	68 (54.8%)	31 (25%)		
Materials for Center Play			0.03	0.85				3.41	0.18
Not Available	2 (33.3%)	4 (66.7%)			3 (50%)	3 (50%)	0 (0%)		
Available	56 (37.1%)	95 (63.0%)			34 (22.2%)	81 (53%)	38 (24.8%)		
Refrigerator for Perishable Items			0.25	0.61				1.35	0.51
Not Available	9 (32.1%)	19 (67.9%)			9 (30%)	16 (53.3%)	5 (16.7%)		
Available	41 (37.3%)	69 (62.7%)			26 (23.6%)	55 (50%)	29 (26.4%)		
Additional Staff Support			1.28	0.26				8.46	0.01 *
Not Available	19 (44.2%)	24 (55.8%)			15 (34.9%)	23 (53.5%)	5 (11.6%)		
Available	36 (34.3%)	69 (65.7%)			19 (17.9%)	54 (51%)	33 (31.1%)		
Funds to Purchase Supplies			2.13	0.14				1.19	0.55
Not Available	19 (45.2%)	23 (54.8%)			13 (29.6%)	20 (45.4%)	11 (25%)		
Available	32 (32.3%)	67 (67.7%)			21 (21.4%)	52 (53.1%)	25 (25.5%)		
Funds for Field Trips			0.50	0.48				5.83	0.05
Not Available	22 (46.8%)	25 (53.2%)			17 (34.7%)	21 (42.9%)	11 (22.4%)		
Available	24 (40%)	36 (60%)			9 (15%)	32 (53.3%)	19 (31.7%)		
FBL Challenges									
Lack of money for additional FBL materials			4.92	0.03 *				3.35	0.19
No	27 (29.4%)	65 (70.6%)			22 (23.9%)	53 (57.6%)	17 (18.5%)		
Yes	32 (46.4%)	37 (53.6%)			16 (22.2%)	34 (47.2%)	22 (30.6%)		
Lack of expertise			0.07	0.79				1.46	0.48
No	49 (37.1%)	83 (62.9%)			33 (24.3%)	73 (53.7%)	30 (22.1%)		
Yes	10 (34.5%)	19 (65.5%)			5 (17.9%)	14 (50%)	9 (32.1%)		
Lack of human resources			1.64	0.20				1.43	0.49
No	39 (33.6%)	77 (66.4%)			26 (22%)	66 (56%)	26 (22%)		
Yes	20 (44.4%)	25 (55.6%)			12 (26.1%)	21 (45.6%)	13 (28.3%)		
Lack of material resources			7.83	0.005 **				0.45	0.80
No	34 (29.8%)	80 (70.2%)			28 (24.4%)	61 (53%)	26 (22.6%)		
Yes	25 (53.2%)	22 (46.8%)			10 (20.4%)	26 (53.1%)	13 (26.5%)		
Other areas higher priority			2.38	0.13				2.64	0.27
No	46 (34.1%)	89 (65.9%)			30 (21.7%)	77 (55.8%)	31 (22.5%)		
Yes	13 (50%)	13 (50%)			8 (30.8%)	10 (38.4%)	8 (30.8%)		
Lack of time			0.56	0.45				0.13	0.94
No	47 (35.3%)	86 (64.7%)			31 (22.8%)	72 (52.9%)	33 (24.3%)		
Yes	12 (42.9%)	16 (57.1%)			7 (25%)	15 (53.6%)	6 (21.4%)		
Lack of knowledge to integrate			0.31	0.58				3.37	0.19
No	49 (35.8%)	88 (64.2%)			29 (20.7%)	76 (54.3%)	35 (25%)		
Yes	10 (41.7%)	14 (58.3%)			9 (37.5%)	11 (45.8%)	4 (16.7%)		
Children not interested in topic			0.08	0.80				1.25	0.54
No	54 (37%)	92 (63%)			34 (22.8%)	81 (54.4%)	34 (22.8%)		
Yes	5 (33.3%)	10 (66.7%)			4 (26.7%)	6 (40%)	5 (33.3%)		
Parents would not support this topic			0.43	0.51				2.64	0.27
No	55 (37.4%)	92 (62.6%)			36 (24.2%)	80 (53.7%)	33 (22.1%)		
Yes	4 (28.6%)	10 (71.4%)			2 (13.3%)	7 (46.7%)	6 (40%)		

Note: * = *p* < 0.05. ** = *p* < 0.01. *** = *p* < 0.001. FBL = food-based learning; PD = professional development; HS = Head Start.

## Data Availability

The data presented in this study are available on request from the corresponding author. The data are not publicly available due to privacy concerns.
